# Editorial: *In vitro* digestion and health benefits of ingredients in plant-based foods

**DOI:** 10.3389/fnut.2023.1255869

**Published:** 2023-07-18

**Authors:** Liang Zou, Peiyou Qin

**Affiliations:** ^1^Key Laboratory of Coarse Cereal Processing, Ministry of Agriculture and Rural Affairs, Sichuan Engineering & Technology Research Center of Coarse Cereal Industrialization, School of Food and Biological Engineering, Chengdu University, Chengdu, China; ^2^Institute of Crop Sciences, Chinese Academy of Agricultural Sciences, Beijing, China

**Keywords:** *in vitro* digestion, plant-based foods, protein, starch, dietary fiber, phenolic compounds, bioaccessibility, processing

The worldwide increase in diet-related diseases has promoted research during the past few decades on the mechanisms of food digestion in the gastrointestinal (GI) tract. Food digestion is a complex process that consists of the oral, gastric, and intestinal phases, in which numerous mechanical, chemical, and enzymatic processes are involved. Several types of digestion models including the *in vivo* (human or animal intervention trials), *in vitro* (INFOGEST) and the *in silico* models have been proposed and used in stimulating the digestion of Plant-based Foods. Compared to *in vivo* methods, *in vitro* simulated GI digestion models have some advantages, such as short in duration, less expensive, and less labor and resource intensive, and also not being burdened with the same ethical restrictions. They are widely employed to study the structural changes, digestibility, and release of food components in the GI tract.

Apart from environmental sustainability and animal welfare considerations, Plant-based foods with several nutritional and health benefits have drawn much interest recently. But their nutritional and health benefits will depend on several factors such as the degree of food processing, the quantity consumed, and one of the most important of these, the amount of nutrients or bioactive compounds finally released and absorbed during GI digestion. Therefore, the key objective of this Research Topic is to elucidate the digestibility and bioaccessibility of dietary ingredients in plant-based foods through application of *in vitro* GI models, and to understand the health effects of their digests.

Finally, a total of four research articles have been published in this Research Topic. The first article finished by Yang et al. investigated the effect of heat treatment on *in vitro* digestion of quinoa albumin. It is obvious that heat treatment decreased the degree of hydrolysis of quinoa albumin and total amino acid content during *in vitro* digestion. Furthermore, the *in vitro* digestion decreased gradually with the increase in treating temperature. Therefore, nonthermal processing technologies including ultrasonication, ultrahigh pressure, and pulsed electric field technologies have been widely used to improve functional, structural properties and digestibility of food protein (1).

Phenolic compounds, which are secondary metabolites found in a diversity of plant materials, exhibit various health benefits, such as antioxidant, anti-inflammatory, antidiabetic, antihypertensive and anticancer. Studies have demonstrated that phenolic compounds are released from food in the digestive system and are further degraded to smaller molecules of metabolites and catabolites which have better bioaccessibility than their original molecular forms (2). In current Research Topic, Luo et al. evaluated the effects of simulated *in vitro* gastrointestinal digestion on antioxidant activities and potential bioaccessibility of phenolic and flavonoid compounds extracted from peels and sarcocarps of three typical *Kadsura coccinea*. The results showed that there are higher levels of total phenolic compounds as well as higher DPPH and ABTS radical scavenging activity in digested fractions compared to indigested fractions. In addition, these three varieties showed relative high recovery rate and moderate bioaccessibility for total phenolic and flavonoid compounds.

Starch is a major energy source of the human diet, starch digestion is closely related to human health, especially in regulating blood sugar (3). To develop an instant powder with anti-diabetic potential, our research group studied the effect of two different extrusion modes including individual extrusion (IE) and mixing extrusion (ME) on the *in vitro* starch digestibility of instant powder which consists mainly of Tartary buckwheat and adzuki bean flour (Zhang et al.). Compared with ME, the instant powder obtained with IE showed lower starch digestibility, lower starch digestion rate constants for the fast step (K_f_), lower estimated glycemic index, higher resistant starch content, and higher α-glucosidase inhibitory activity, indicating that the Tartary buckwheat/adzuki bean instant powder produced by IE could serve as an ideal anti-diabetic food resource.

Numerous epidemiological and interventional studies have demonstrated that dietary fibers have important associations with the development and management of various diseases, such as reduce risk of cardiovascular disease, diabetes, and some cancers (4). However, intake of dietary fibers can induce GI symptoms which limited the utility of dietary fiber. The fermentable oligo-, di-, monosaccharides and polyols (FODMAPs), which include fructans and galacto-oligosaccharides, lactose, fructose, and sugar alcohols, has emerged as key contributors to increased GI symptoms (5). Therefore, Guice et al. investigate the efficacy of a food-grade, microbial inulinase preparation with companion invertase activity on several dietary fructan-rich substrates in the static INFOGEST *in vitro* digestion simulation. The *in vitro* digestion data suggested that the use of microbial inulinase as an exogenous enzyme could reduce dietary fructan-type FODMAP exposure.

To conclude, the present Research Topic provides several examples which *in vitro* simulated GI digestion models were applied to characteristic the properties of digestibility, function and structures of protein, phenolic compounds, starch and dietary fiber from plants. To better simulate the real gastrointestinal digestion of the nutrients or bioactive compounds from plant-based foods, more work is needed to improve or develop *in vitro* GI models. Moreover, the health benefits of the digestive products of plant-based foods should be further studied in the future.

## Author contributions

All authors listed have made a substantial, direct, and intellectual contribution to the work and approved it for publication.
